# Creation Process of the Digital Platform to Foster Healthy and Active Aging: *enbuenaedad*

**DOI:** 10.3389/fpubh.2019.00022

**Published:** 2019-02-19

**Authors:** Sandra Pinzón-Pulido, Mónica Padial-Espinosa, Luz López-Samaniego, Bibiana Navarro-Matillas, Pilar San Juan-Lozano, Juan Manuel Espinosa-Almendro, Josefa Ruiz-Fernández, Francisco Garrido-Peña

**Affiliations:** ^1^Andalusian School of Public Health, Granada, Spain; ^2^Regional Ministry of Health of Andalusia - FPS, Seville, Spain; ^3^Andalusian Health Service, Primary Health Care Center El Palo, Málaga, Spain; ^4^Department of Criminal Law, Law Philosophy, Moral Philosophy and Philosophy, University of Jaén, Jaén, Spain

**Keywords:** public health, active and healthy aging, co-thinking, ICT, health promotion

## Abstract

Andalusia is a region in the south of Spain with 8,4 million inhabitants of which 1,3 million are over 65 years old. Andalusia has been recognized as Reference Site by the European Commission within the European Innovation Partnership on Active and Healthy Aging. The Regional Ministry of Health of Andalusia has put in place strategies to promote healthy and active aging. One of these strategies is *enbuenaedad*, a digital platform which main aim is to foster active and healthy aging. The target audience is people over 55 years old, caregivers of older adults, as well as health and other key professionals who work with this population. Content sections are inspired in the three pillars of the World Health Organization (WHO) policy framework for active and healthy aging: health, participation, and security, but introducing an additional one which is lifelong learning. One of the strengths of this platform is the creation process. Using a co-thinking design, all target groups get voice under the umbrella of empathy and are empowered by providing support, training, knowledge, and best practices. For its development, dissemination, maintenance, and improvement, the project advocates the unavoidable participation of key stakeholders representing all sectors involved: The Senior Council of Andalusia; Primary Health Care professionals; local authorities; *Guadalinfo* agents; Permanent Adult Education; and Active Participation Centers. Quantitative and qualitative data obtained within the process support this project. Since its launching, 10,779 users have registered to the platform with more than 157,000 visits. Focusing on WHO four pillars on active and healthy aging *Enbuenaedad* is based on, preliminary results show effectiveness regarding participation and social interaction. Furthermore, achieving high participation coverage is a necessary but not sufficient input to the provision of adequate approach to older people. More comprehensive evaluation of the four pillars must be taken to ensure a holistic approach. A challenge is a cooperation between three traditionally independent sectors, cooperative work between health, social services, and education is crucial for the future sustainability of this intervention.

## Introduction

Andalusia is a region in the south of Spain with 8.4 million inhabitants. With 1.3 million people over 65 years old. Spain's aging rate is one of the highest among advanced economies and continues to increase steadily. By 2040, Spain will have the longest life expectancy in the world, according to global statistics (1).

This longevity revolution demands effective political action. It is essential to research and innovate to develop public health policies that empower older people (2–4). In the last decade, Andalusia has put in its agenda many initiatives to promote active and healthy aging. The European Innovation Partnership for Innovation on Active and Healthy Aging (EIPonAHA) of the European Commission (5) has awarded Andalusia as reference site on active and healthy aging. All reference sites follow the Quadruple Helix-Based Model for Active Aging. This model involves government authorities, civil society, academia, and business for the development of policies on active and healthy aging (6).

## Background and Rationale

Longevity implications on the lifespan of the heterogeneity population of Andalusia are the starting point of this project (7).

Based on the literature review, the community needs and previous theoretical frameworks, we have developed a conceptual framework. It maps out the potential role of information and communications technologies (ICTs) as resources to empower older people so that they can manage their health condition and well-being (8–10), as well as to facilitate healthy decision-making and connect older people with healthcare networks (11). Usefulness of ICTs depends, to a large extent, on its capacity to adapt to the functional, social, and behavioral features of the users, as well as to their needs and preferences (8–11). Older people have the lowest level of ICT adoption (12). The success of e-inclusion in this population group seems to rely on both the digital literacy policies and the development of suitable devices and software (13).

The methodology used for the design, implementation, and evaluation of this study is based on the Quadruple Helix-Based Innovation Model for Active Aging, and on a method that enables effective participation of all agents involved in the process (14).

## Essential Elements of the Intervention

This section identifies the essential elements of the intervention. These are not mutually exclusive categories; indeed, more effective intervention is likely to result when a holistic approach is addressed across all these dimensions.

### Identifying the Target Population

**Figure 1:**
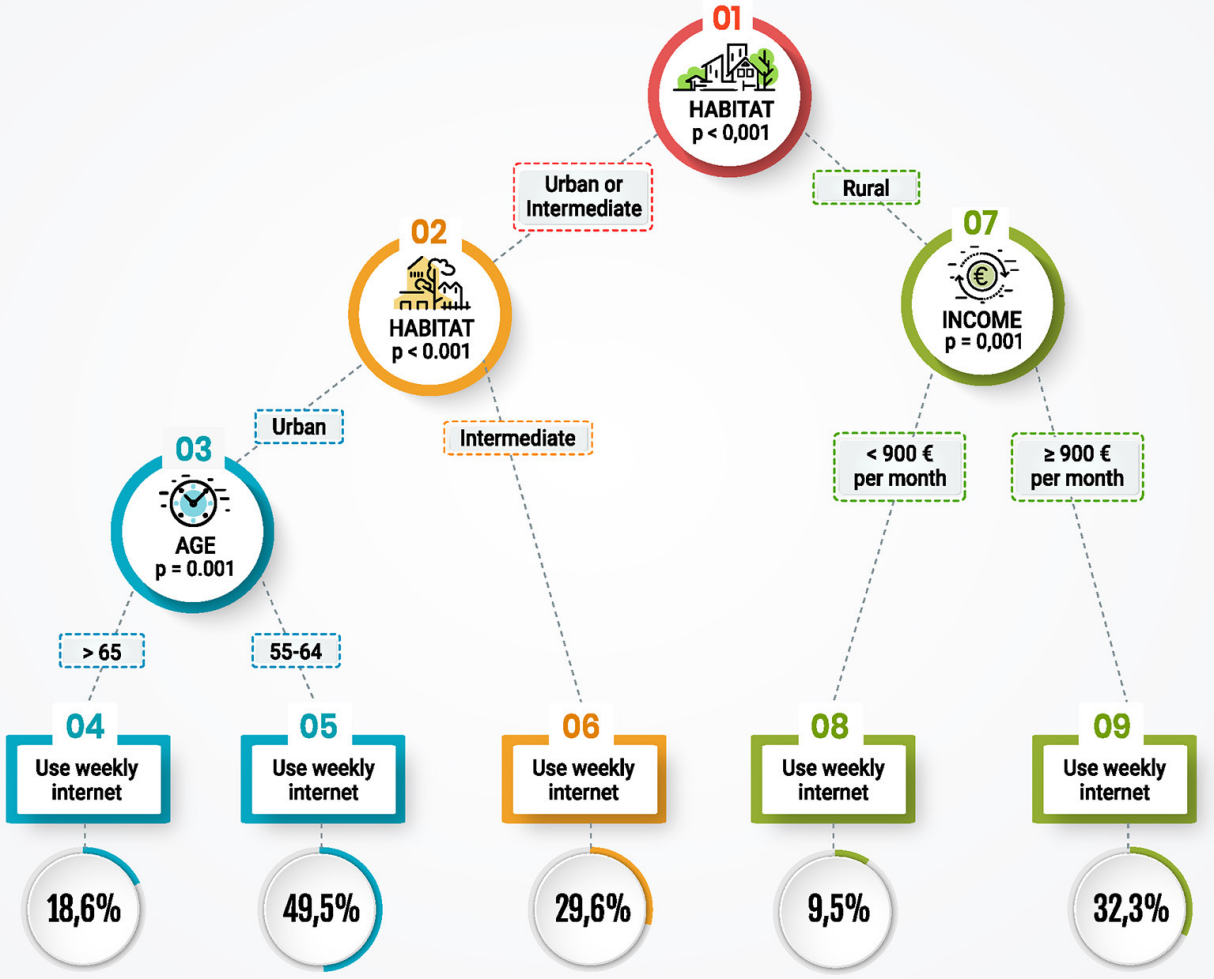
Older people' segments according to weekly Internet use.

A survey on ICT usage was carried out by the research team in collaboration with the University of Seville. In public health evaluations, the units of analyses are usually population groups, rather than individuals. In this project units are two groups: one group of people between 55 to 64 years old; and another one aged between 65 and 74. Main results were that 57% of people between 55 to 64 years old use the Internet on a weekly basis, while there was a significant reduction in the prevalence of the second group. Figure 1 shows the population segmentation of internet users. These findings contributed to people between 55 to 64 years be included as the target population, and to ensure a strategy oriented to reduce the disparities observed by income or habitat.

### Older Adults-Centered Approach

In-depth interviews, focal groups, and community forums provided key elements to take into consideration their vision to the development of digital solutions (15, 16). Heterogeneity in ICT adoption was a fact, and so was their interests on the opportunities they demanded and prioritized regarding healthy and active aging. A permanent testing strategy was established by the National Confederation for Active Older Adults and the En Activo Association to ensure that developed prototypes met the expectations and preferences of older adults.

### Effective Participation of the Stakeholders

**Figure 2:**
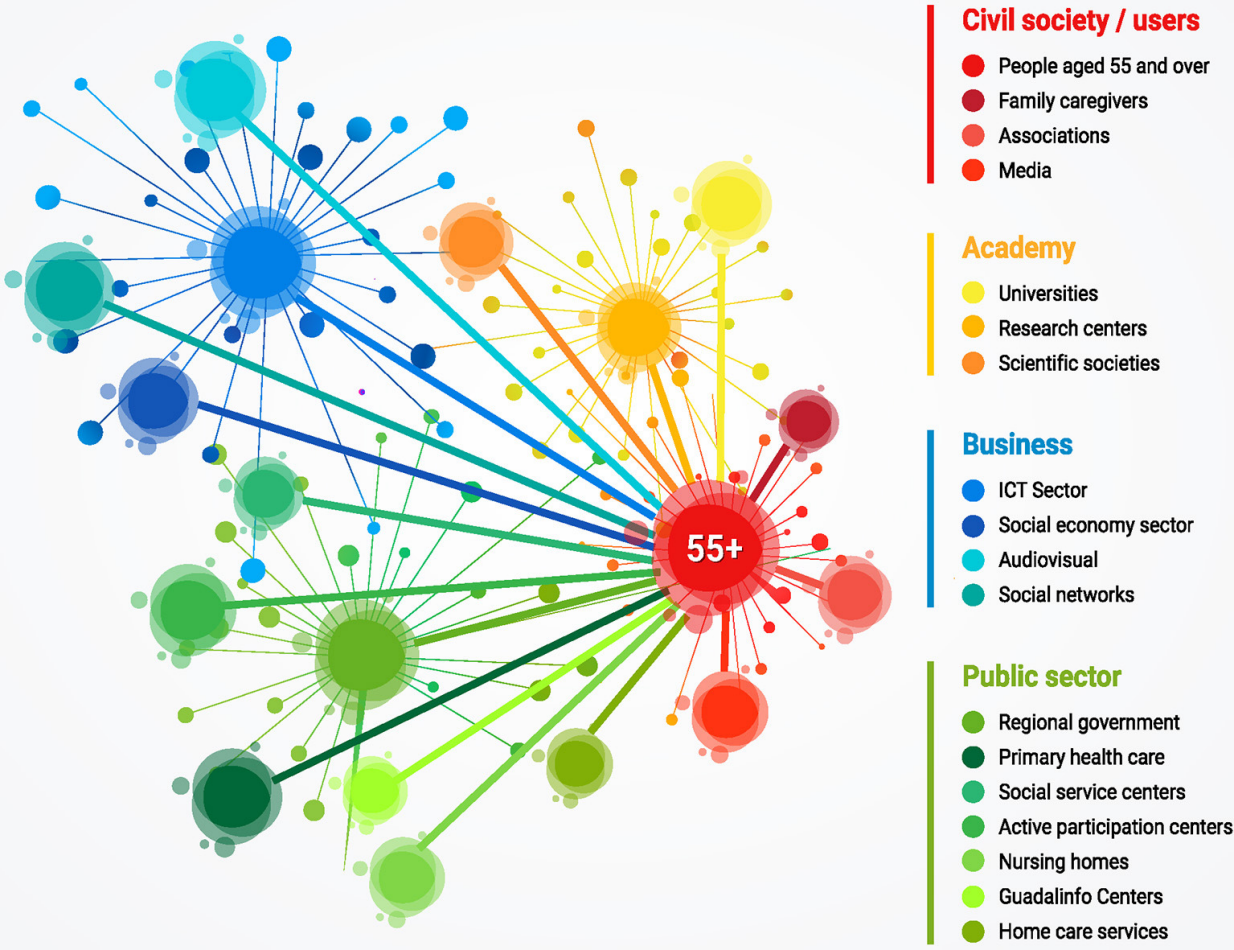
Stakeholders involved in the promotion of active and healthy aging.

Figure 2 lists the different stakeholders -aligned with the four Quadruple Helix-Based Innovation Model on Active Aging.

### Cooperative Design

**Figure 3:**
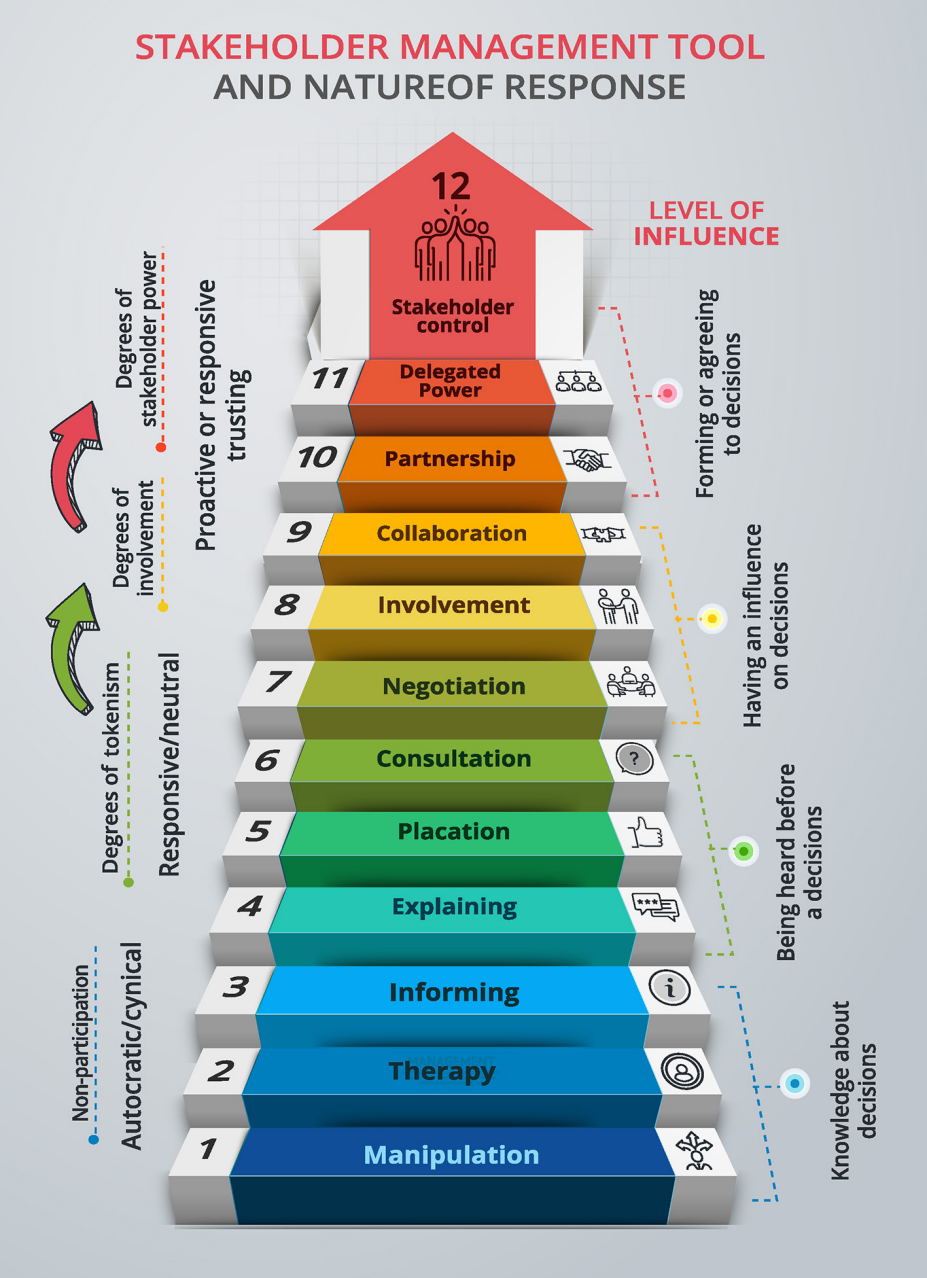
From the consultation and negotiation to the delegation of power.

The first year of the project was devoted to the creation of the website and the development of the first digital content. The method used was the collaborative design known as design thinking (17). Literature review and synthesis techniques, qualitative research techniques for data collection and analysis and consensus group techniques were used, both for the design and the testing of the products (15). Contents and format of the web platform and the digital solutions were determined upon the expectations expressed by older people involved in the design process (16). The collaborative constructions of these products contributed to the rapid acceptance of the platform by the target population. Figure 3 shows “the citizen participation ladder,” a step-by-step approach to the decision-making process.

### Combating Inequality

Gender, age, ethnicity, socio-economic status, functional capacity, and habitat are, among others, factors that can be significant in how ICTs are accessed, owned, or used (18). The intervention developed here presents specific strategies to address these factors.

## Methods

### Intervention Design

Action research approach using qualitative and quantitative research techniques (14, 15).

### Target Population

Women and men, aged 55 and over, caregivers, association representatives, healthcare, education, and social care professionals, authorities from public institutions and administrations, private companies, entities from the economic sector and other stakeholders involved in the promotion of active and healthy aging in Andalusia.

### Methodology

The design thinking method was used as an essential tool to simplify and humanize the process of designing the digital solutions that are the object of the project and achieve their adoption by the target population (16).

## Description of the Case

Step 1: Develop the digital platform.

The functional architecture of the platform was designed by the target population themselves. In this case, we use community and professional forums to design content and formats. Needs and expectations became technical requirements. A card-sorting technique was used to determine the functional architecture of the portal. A prototype was developed and tested with the user population. Contents are organized in the four pillars of active aging: health, security, participation, and lifelong learning. Each section incorporates audio-visual content produced under a collaborative design framework. All platform users can share resources, experiences, activities, etc. The name “Enbuenaedad” which translated into English would be “in a good age,” is also the result of a consensual agreement. The platform incorporates an instant messaging service. There is a specific space for health professionals. Easy-to-follow tutorials are available to facilitate navigation and interaction with the platform. Also, there are links to connect to social networks. More than 157,000 visits during the first 6 months.

Step 2: Develop the instant messaging service.

Personalized messaging service for the promotion of healthy aging. These messages, designed by health professionals and tested by older people, aim to encourage the acquisition of healthy habits. The system incorporates tools to assess the effectiveness of the messages and their contribution to autonomy in decision-making. By now 8,089 older adults (4,003 female and 4,086 male) are registered. More than 77,000 thousand messages about healthy and active aging have been sent.

Step 3: Determine the dissemination plan.

In 2017, the corporate image of the project was developed, a slogan was designed, and the key ideas for all communication actions were determined. In 2018, 20 forums were undertaken; 5 scientific publications were produced; 10 communications were presented to scientific conferences; a Facebook page was launched (with around 20,000 thousand interactions during the first semester). This is an ongoing process over time and requires sustained efforts.

**Figure 4:**
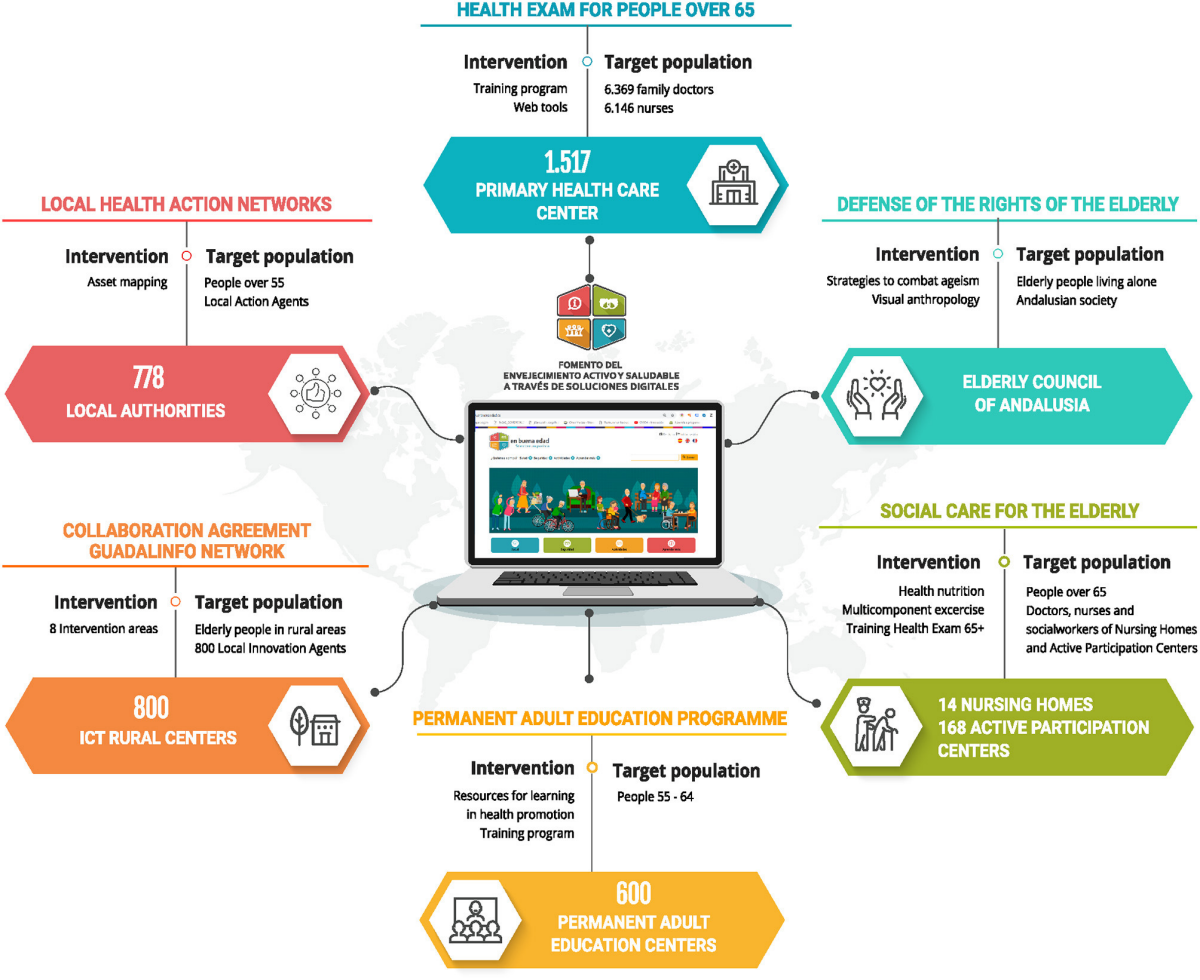
Key stakeholders, new functionalities and new contents.

The success of the dissemination of the platform will be maximized if older people is reached at a variety of locations (Figure 4).

### Primary Care Centers

The program Health Examination for People over 65 years was updated in 2017. It was initially implemented in Primary Care in 2006. 839,019 patients were registered by 2015: 61% autonomous, 24% frail, and 18% dependent. This update aimed to include promotion and prevention interventions related to active and healthy aging; the early detection of frailty and risk of falls; develop interventions aiming at the recovery of functional capacities; and the optimization of residual capacities of dependent people. To implement the new programme in the 1,517 primary care centers of the public health system of Andalusia a communication and training strategy was designed. Six thousand Three Hundred and Seventy-Nine doctors and 6,146 nurses are responsible for the healthcare of 1.3 million adults over 65 in Andalusia. The design thinking methodology was used to develop the digital contents. Audio-visual production companies and primary care medical and nursing professionals worked on the creation of the program. Three training itineraries were designed and 10 online continuing education activities. All these contents are currently at the pilot testing phase. Once this phase is over, the next step would be conducting massive online open courses (MOOC) in an attempt to reach the entire group of primary care professionals. 18,414 men and 24,072 women over 65 enrolled during the first 4 months of the program. The program is included as part of the content of the platform *Enbuenaedad*.

### Local Health Action Networks

The strategy for identification and mapping the resources and assets for health has been led by the Andalusia Local Network Strategy for Action in Health (RELAS). This network covers 20.4% of the town councils, and 43.2% of the Andalusian population, through conventions signed between the Regional Ministry of Health of Andalusia and 155 municipalities from the 8 Andalusian provinces. Using the design thinking techniques, the aim is to work with older adults, RELAS health promoters, monitors, representatives from the voluntary sector, technical staff from the town councils responsible for leisure, diet, and physical activity programs, etc. (19).

### Rural Areas

The study of population segmentation according to the frequency of the use of the Internet, highlighted the existing digital division, more acute in older adults with less favorable socio-economic conditions, living in rural environments. To get this population involved in the design and the use of solutions to promote active and healthy aging, the centers of the Guadalinfo Network were identified as a key stakeholder. Guadalinfo is a network of public centers in Andalusia. Guadalinfo centers are located in municipalities with less than 20,000 inhabitants and the most disadvantaged areas of cities. These centers promote equal opportunities in the Access to ICT. The network has around 800 physical centers and 800 local innovation agents working with over a million service users. After first contacts and preliminary interviews, a collaboration agreement was signed. A first forum was organized, and all local innovation agents from the province attended. Their expectations were collected in this meeting. A survey was carried out, the results of this survey pointed out eight priority areas: healthy eating, physical activity, skin care, constipation, health control, polypharmacy, home security, and grandchildren care. A design group was created engaging territorial dynamizers from Guadalinfo and health promotion technical staff. Also, the Local Innovation Agents were trained in the use of the web platform. Training sessions were conducted in two different municipalities to ensure maximum participation. To enable communication and participation of all agents during the solutions design process, a model of the bulletin was proposed to be disseminated via the *Enbuenaedad* platform and Guadalinfo web portal.

### Permanent Adult Education Program

These centers, with a total amount of 600 in Andalusia, offer formal training activities for adults and non-formal training schemes, offering, among others, training in the use of ICT, languages, entrepreneurial culture, environmental and cultural heritage, healthy lifestyle, etc. Within this framework of non-formal training schemes, a line of collaboration has been established with the Regional Ministry of Education of Andalusia to promote active and healthy aging. Following the Design Thinking method, face-to-face meetings were held during 2017 and 2018. In the light of the needs expressed by teachers and pupils from the adult permanent education centers, permanent learning materials should focus on: balanced diet, physical activity, security at home, safety in the environment, memory, elder abuse prevention, emotional well-being, affectivity, and personal development.

### Active Participation Centers

There are 14 Residential Homes and 168 Active Participations Centers (APCs) in Andalusia. The aim to include such centers is that there is no structured intervention ensuring a balanced diet and physical activity adapted to the functional capacity and frailty risk of the older people users of these centers. The need to address this issue is based on the high rates of malnutrition and undernutrition among older adults. Malnutrition affects 70% of older adults that live in residential homes (20). Thirty Percent of those adults are undernutrition, and 50% are at risk for undernutrition (21). With regards to physical activity, previous studies have shown that it contributes to the happiness of older adults and that it directly influences the perceived importance of interpersonal relationships, self-concept, and personal independence (22). On the other hand, an intervention with multicomponent exercise reverts frailty and improves cognitive status, emotional relations, and social networks in weakened older adults (23, 24). In this intervention, the project proposes six areas of intervention through the digital platform: (1) Eat as at home. This intervention uses mealtimes to promote the autonomy of the institutionalized older adults and prevent undernutrition; (2) A reformulation of the dining service to reinforce a balanced diet and prevent undernutrition; (3) Create catering services for weakened older adults through APC volunteering service; (4) Multicomponent physical activity program that includes muscular strength, balance and flexibility, and aerobic endurance exercises; (5) Professional Training Program to ensure a balanced diet and the practice of physical activity in residential homes and APCs; and (6) MOOC Program on a balanced diet and physical activity for older adults.

### The Senior Council of Andalusia

The senior Council of Andalusia is responsible for, among others, promoting and protecting the interests of older people in public and private entities; encouraging active participation of older population by acting as interlocutor of the group before public authorities; and promoting the association by providing the technical support required by the organizations of older adults to encourage their participation in society. The web platform has been presented in conferences organized by the Provincial Council for older adults and other local stakeholders. Also, presentation and training sessions on the use of the platform are being conducted in the eight Andalusian provinces, organized by the Territorial Delegations and the Provincial Councils for older adults. The fundamental contribution of Provincial Councils lies in the detection of needs, desires, and expectations of older adults they represent. Issues closely related to the guarantee of the rights of older adults and the fight against stigma on the grounds of age or inequalities will be developed in collaboration with these stakeholders. To address these issues, the 2018-202 budget now includes the elaboration of a map of legacy, an initiative awarded by the World Health Organization (WHO) for combating ageism, and an approach to the unwanted loneliness through visual ethnography.

Step 4: Establish the monitoring and evaluation system.

A conceptual model has been developed for such issue with not conclusive results by the time being due to the short period to be evaluated. Also, we are testing an index for the Self-assessment of Healthy and Active Aging.

## Discussion

*Enbuenaedad* is the first attempt to foster healthy and active aging through a digital platform in Andalusia. On the one hand, Gustafson et al. (25) highlight the importance of taking the target population into account when developing ICTs on older adults. On the other hand, Le Rouge et al. (9) concluded that user profile information is important data for effective ICTs design. Both ideas are involved in the methodology to develop this project. Older people are essential advocates of their health interests and concerns. They need to be recognized with respect and given a voice when developing policies and plans using ICTs. The results achieved so far demonstrate the positive effect of this method.

Part of the success of the platform has to do with adapting to user expectations. Isakovic et al. (26) advocate for adaptations in size, visibility, comprehensibility of buttons and symbols, as well as the use of tutorials and additional explanations improve the adoption of ICT by the older population. One of the main concerns of this intervention has been to reach the entire target population, with a particular focus on the use of inclusive ICT for people with disabilities. In this intervention, community and professional forums helped to identify specific structural elements of the platform. For example, color contrast for people with low vision, or colored backgrounds to improve the readability.

Demiris et al. (12), point out that older people need adequate and personalized training for their adaption to ICT. Our project contributes to such training through tutorials and with the help of stakeholders.

Last but not least, this project aims well-being promotion in older people. According to Demiris et al. (12), ICTs can provide support and facilitate a comprehensive welfare benefits advice aimed at maximizing decision-making and network accessibility. Le Rouge et al. (9) underlines that older people able to take an active role in their health feel more empowered. To this conclusion, we add the participation pillar. For older people to be social is essential, it is empowering for older people to be social and participative.

## Lessons Learned

*Enbuenaedad* can become an effective advocate for the needs of older people by constituting a strong via for their interests and concerns.

*Enbuenaedad* development and dissemination require a multidisciplinary approach. There has been increasing recognition of the need to promote active and healthy aging through the use of ICT.

It is important to transmit stakeholders and sectors a clear understanding of their responsibilities. All the stakeholders identified earlier need to be trained to provide an integrated activity across different institutions. They can play an essential role in implementing healthy and active aging. Ideally, all stakeholders should participate in planning to take advantage of the unique skills and experiences of each sector.

It is necessary to have the support of graphic designers to establish a powerful audio-visual communication.

The creation of prototypes is an integral part of our innovation process. We build prototypes to think and learn.

Orientation to action. It is about doing, more than thinking and meeting.

One challenge is to understand the experience of the users. We use qualitative techniques to design the solutions and test their emotions and perceptions. Different experiences and points of view are considered a plus.

One limitation lies in approaching the most vulnerable populations and those with limited access to ICT.

In relation to training, the platform is not sufficiently adapted to the needs of the users. It is necessary to design an App to support this limitation. In the same line, the platform is not enough adapted to the needs of health, social services and education professionals, and caregivers. An App will be developed for that issue.

Focusing on WHO four pillars on active and healthy aging *Enbuenaedad* is based on, preliminary results show effectiveness regarding participation and social interaction. Furthermore, achieving high participation coverage is a necessary but not sufficient input to the provision of adequate approach to older people. More comprehensive evaluation of the four pillars must be taken to ensure a holistic approach. A challenge is a cooperation between three traditionally independent sectors, cooperative work between health, social services, and education is crucial for the future sustainability of this intervention.

## Ethics Statement

This study was carried out in accordance with the recommendations of the World Medical Association's Declaration of Helsinki. The protocol was approved by the General Secretariat of Public Health of the Regional Ministry of Health and the Research Ethics Committee of Jaen of the Andalusian Network of Research Ethics Committees. Written informed consent has been documented in a form provided by the Andalusian School of Public Health. This informed consent indicates how identifiable data are used and protected. The form includes a section in which participants are encouraged to ask any questions and to ensure they are comfortable before they sign the consent form. The anonymity of the participants and the confidentiality of the data have been guaranteed according to the Law 14/2007, of Biomedical Research of Andalusia and the Regulation (EU) 2016/679 of the European Parliament and of the Council of 27 April 2016 on the protection of natural persons with regard to the processing of personal data and on the free movement of such data, and repealing Directive 95/46/EC (General Data Protection Regulation). We applied the concept of an “identifiable natural person” to ensure that it was not possible to establish the link between the data and the study subjects.

## Author Contributions

SP-P, LL-S, JE-A, JR-F, and FG-P, make substantial contributions to conception and design of the study and wrote the first draft of the manuscript and wrote sections of the manuscript. MP-E, BN-M, and PS, performed the data analysis. All the authors revised the paper critically for important intellectual content; and gave final approval of the version to be submitted.

### Conflict of Interest Statement

The authors declare that the research was conducted in the absence of any commercial or financial relationships that could be construed as a potential conflict of interest.
